# Quantifying peatland land use and CO_2_ emissions in Irish raised bogs: mapping insights using Sentinel-2 data and Google Earth Engine

**DOI:** 10.1038/s41598-024-51660-0

**Published:** 2024-01-12

**Authors:** Wahaj Habib, Ruchita Ingle, Matthew Saunders, John Connolly

**Affiliations:** 1 Discipline of Geography, School of Natural Sciences, Trinity College Dublin, Dublin, Ireland; 2 Discipline of Botany, School of Natural Sciences, Trinity College Dublin, Dublin, Ireland; 3 Water Systems and Global Change Group, Wageningen University, Wageningen, The Netherlands

**Keywords:** Environmental impact, Climate-change adaptation, Geomorphology

## Abstract

Ireland has > 50% of the EU’s ocean-raised bogs; however, degradation through land-use activities has transformed them from carbon (C) sinks to sources. Given their significant role in climate mitigation, it is essential to quantify the emissions resulting from land use degradation of these ecosystems. A seven-class land-use classification system for Irish peatlands (LUCIP) was developed and mapped using Sentinel-2 imagery, random forest machine learning and Google Earth Engine. The results revealed that agricultural grassland comprised 43% of the land use on raised bogs, followed by, forestry (21%), cutover (11%), cutaway (10%) remnant peatlands (13%), waterbodies and built-up ~ 1% each. The overall accuracy of the map was 89%. The map was used to estimate CO_2_ emissions for four classes constituting 85% of raised bogs: cutover, cutaway, grassland, and forestry using the IPCC wetlands supplement and literature-based emission factors, we estimated emissions at ~ 1.92 (± 1.58–2.27 Mt CO_2_-C-yr^−1^) and ~ 0.68 Mt CO_2_-C-yr^−1^ (± 0.44–0.91 Mt CO_2_-C-yr^−1^) respectively. This is the first study to spatially quantify land use and related emissions from raised bogs. The results have revealed widespread degradation of these globally rare habitats, making them net emitters of CO_2_. The map is vital for the conservation of these ecosystems through restoration efforts, and the methodology can also be applied to other regions with similar peatland land use issues.

## Introduction

European peatlands cover approximately 60 million ha^1,2^, and a substantial proportion of this has been degraded by unsustainable land use practices, such as agriculture, forestry, and peat extraction. Consequently, almost 44% can no longer accumulate peat^3^. Tanneberger et al.^4^ indicated that degraded peatlands in the European Union (EU) represent half of the globally degraded peatlands. In Ireland, peatlands cover ~ 1.46 million ha (21% of Ireland) and consist of two types of bogs: blanket bogs (~ 900,000 ha) and the globally rare Oceanic raised bog (~ 530,000 ha)^5,6^. It is estimated that they store between 60 and 75% of the national Soil Organic Carbon (SOC) stock^7–9^ but land use practices have led to the degradation of about 95% of these peatlands^6,10–13^.

These land use activities harm the hydrological and ecological functioning of peatland ecosystems, turning them from a net sink to a source of C^14^. It also results in a consistently lowered water table, which accelerates peat decomposition, releases dissolved organic carbon (DOC) and particulate organic carbon (POC) and alters carbon and greenhouse gas (C and GHG) fluxes^15,16^. Hence, these ecosystems, which in their natural condition are significant C stores and are crucial to the prevention of natural disasters such as landslides, fires, and floods^17^, become susceptible to them^18–20^. Despite this, Irish peatlands still contribute to various hydrological and ecological functions and cultural, and socio-economic values^6,21^.

The land use practices on peatlands in Ireland are similar to those in Europe, including drainage for industrial and domestic peat extraction, agriculture (mostly grasslands), afforestation and infrastructure development (roads, wind farms, airports etc.)^12,22,23^. As a result, it is estimated that less than 1% of raised bogs in Ireland are actively forming peat as of 2017^24^. This is particularly due to the intensification of land use through industrial peat extraction, afforestation and agriculture, which is a relatively recent development (since the 1940s), but has caused more damage in a short period of time^22^. The establishment of Bord na Móna (BnM), a semi-state-owned company, in 1946 for industrial peat extraction had a notable impact on raised bogs. Approximately 90% of the BnM landholdings are situated on raised bogs, and the majority of those (~ 90%) have been drained and/or opened for peat extraction^62^. BnM ceased extraction activities in 2021^25^, however, there are still several medium-sized companies engaged in industrial peat extraction, including Harte, Klasmann-Deilmann, Bulrush, Clover, Erin, and Westland, as well as ~ 30 other small producers^11,14^. Coillte, a semi-private afforestation company established in 1989, has afforested approximately 31,000 ha of raised bogs. The provision of grants through the Common Agricultural Policy (CAP) for the reclamation of raised bog edges for agriculture has also contributed to the intensification of land use in these ecosystems. However, spatially explicit data on the extent of these activities (especially non-BnM industrial activities) and domestic peat extraction activities is non-existent^26^. The absence of such data prevents accurate quantification of emissions from managed peatlands and hinders the formulation of effective conservation strategies.

As a signatory to the United Nations Framework Convention on Climate Change (UNFCCC), Ireland has an obligation to report and account for its GHG emissions and removals and meet its emission targets for Land Use, Land Use Change, and Forestry (LULUCF), including wetlands, as outlined in the EU's 2030 climate change framework^27^. Several studies have been conducted on Irish raised bogs at small spatial scales to gain insights into the effect of land use conversion activities on C and GHG dynamics, such as losses through land, atmospheric, and fluvial emissions^28,29^. These local-scale effects can have regional and global consequences; yet information on land use status and associated emissions from these ecosystems remains poorly understood at the national, regional, and global levels^30^. To propagate local-scale emission measurements to a national scale and accurately account for peatland emissions, spatially explicit information on peatland land use is essential^14,26^. Andersen et al.^31^ emphasise the importance of land use/cover data in understanding degradation and restoration. However, national and global land-use/cover maps often exclude activities such as peat extraction, forest and abandoned peat extraction sites^4^. This makes these datasets inadequate for understanding degradation through land use, monitoring conservation activities (rehabilitation, restoration, and rewetting), and assessing C and GHG emission dynamics.

To report emissions from wetlands, the Intergovernmental Panel on Climate Change (IPCC) has proposed a system consisting of three tiers: Tier 1 (T1) includes default EF and is specifically for wetlands and Tier 2 (T2) EFs are country specific and based on the emission data from case studies. The Tier 3 (T3) EFs use more complex and dynamic models. The default T1 EFs are derived from limited data available from sites with different geographical, climatic, and ecological conditions. Ireland reports emissions from peatlands based on T1 EFs except for industrial peat extraction sites and forests on drained peatlands, where country-specific EF values are being used^32^. However, the reliance on coarse spatial resolution (25 ha) CORINE (Co-ordinated Information on the Environment) land cover data and non-spatially explicit information for reporting purposes make the national emission estimates less accurate. Given these limitations, it is essential to develop peatland-specific land use maps that can be used for more accurate upscaling of emissions from site to regional, national, and global scales using IPCC methods. Furthermore, these maps are vital for supporting conservation activities, sustainable land management practices, and policy-related decision making.

This study aims to map the much-needed spatial distribution of land use on raised bogs in Ireland and quantify the corresponding C and GHG emissions^5,33,34^. The spatial extent of raised bogs was based on the DIPMv2 (Derived Irish Peat Map version 2), which is the latest peatland extent map integrating data from multiple sources^5^. Spatially explicit information on land use can be obtained using remote sensing methods. However, the persistent cloud cover inherent to the temperate maritime climate of Ireland poses a challenge to the acquisition of frequent cloud-free optical remote sensing images^36^. Consequently, it is particularly difficult to map national-scale land cover and land use. Previous attempts to map land cover at a national scale in Ireland have encountered similar difficulties and full coverage has not been achieved^36–39^. Connolly^12^ successfully mapped industrial, grassland and forestry on peatlands in Ireland, including raised bogs, but was unable to map domestic peat extraction, because of medium spatial resolution of the data (23 m) and cloud cover issues.

Google Earth Engine (GEE), a planetary-scale cloud computing platform^40^, integrates freely available high-resolution satellite imagery such as Sentinel-2, as well as machine learning algorithms such as random forest. By leveraging a vast archive of satellite imagery and employing temporal mosaicking functions within GEE, it is possible to obtain cloud-free imagery with wall-to-wall coverage and use it to map land use in areas where cloud cover impedes optical remote sensing^41–43^. This approach was used in this study to obtain cloud-free imagery by mosaicking annual images acquired for three years i.e., from 1st January 2018 to 31st December 2020. As a result, the first national-scale land use map of raised bog with seven classes was developed. The classes were derived from the Land Use Classification of Irish Peatlands (LUCIP), which was developed for this study through discussions with various stakeholders including NPWS (National Parks and Wildlife Service), BnM, and an ecologist. The classes in LUCIP includes cutover, cutaway, grassland, forestry, remnant peatlands, water bodies, and built-up areas. The map enabled the assessment of the spatial extent of land use in raised bogs. It is further used to estimate CO_2_ emissions using site-specific EFs from case studies in Ireland^26^, which are then presented along with IPCC T1 EFs. This work will provide better insight into C dynamics at the site scale, with an emphasis on the importance of high-resolution maps for area estimation and country specific EFs.

## Material and methods

### Study area

The study area for this research was based on the spatial extent of raised bogs derived from the DIPMv2^5^. Raised bogs are predominantly situated in the midlands of Ireland and are a distinctive feature of this inland region. They cover ~ 530,000 ha of the surface area and constitute ~ 35% of the total peatland area in Ireland and 8% of the total land surface area. Irish raised bogs represent more than 50% of oceanic-raised bogs in the EU^35^.

### Satellite image data

Copernicus Sentinel-2-MSI (Multi-Spectral Instrument) optical remote sensing satellite images were used in this study and the images were acquired by Sentinel 2- A and B. The Sentinel-2 sensor has 13 spectral bands, 10 of which were used in this study: Red (R), Green (G), Blue (B), Near Infrared (NIR) and Narrow NIR, three of the Vegetation Red Edge, and two of the Short-Wave Infrared (SWIR)^44^. Table 1 shows the spectral and spatial resolutions of the bands used in this study. The RGB and NIR bands have a spatial resolution of 10 m while the rest were resampled from 20 to 10 m. The sensor has a swath width of 290 km^44^.

**Table 1:** Characteristics of sentinel-2 bands^45^.

Bands	Central wavelength (µm)	Spatial resolution (m)
Band 2—Blue	0.490	10
Band 3—Green	0.560	10
Band 4—Red	0.665	10
Band 5—Vegetation red edge	0.705	20
Band 6—Vegetation red edge	0.740	20
Band 7—Vegetation red edge	0.783	20
Band 8—NIR	0.842	10
Band 8a—NNIR	0.865	20
Band 11—SWIR	1.610	20
Band 12—SWIR	2.190	20

GEE provides the Sentinel-2-MSI Level-2A (L2A) image archive dating back to March 2017. This study used the atmospherically corrected products:L2AS2_SR (Sentinel-2 Surface Reflectance). They are corrected for atmospheric, slope and adjacency effects. L2A processing is based on a two-step process i.e., (1) scene classification (SC), which is used to derive a pixel classification map for vegetation, snow, soil, cloud shadows and cloud and (2) Sentinel-2 Atmospheric Correction (S2AC) which is used to convert the Top of Atmosphere (TOA) reflectance to BOA (Bottom of Atmosphere) values. The S2AC process is based on the library for Radiative transfer (libRadtran) model^46,47^. It provides cloud, haze, and water removal methods. To enhance the classification process, the vegetation and water indices were calculated and included as additional bands. The Normalised Difference Vegetation Index (NDVI) with values ranging from 0.1 to 1 indicates the presence and health of vegetation^48^ (Eq. 1), while the Normalised Difference Water Index (NDWI) with values ≥ 0.5 indicating the presence of open water^49^ (Eq. 2).

1 $$NDVI=\frac{NIR-Red}{NIR+Red}$$

2 $$NDWI=\frac{NNIR-SWIR1}{NNIR+SWIR1}$$

### Image classification methods

**Figure 1:**
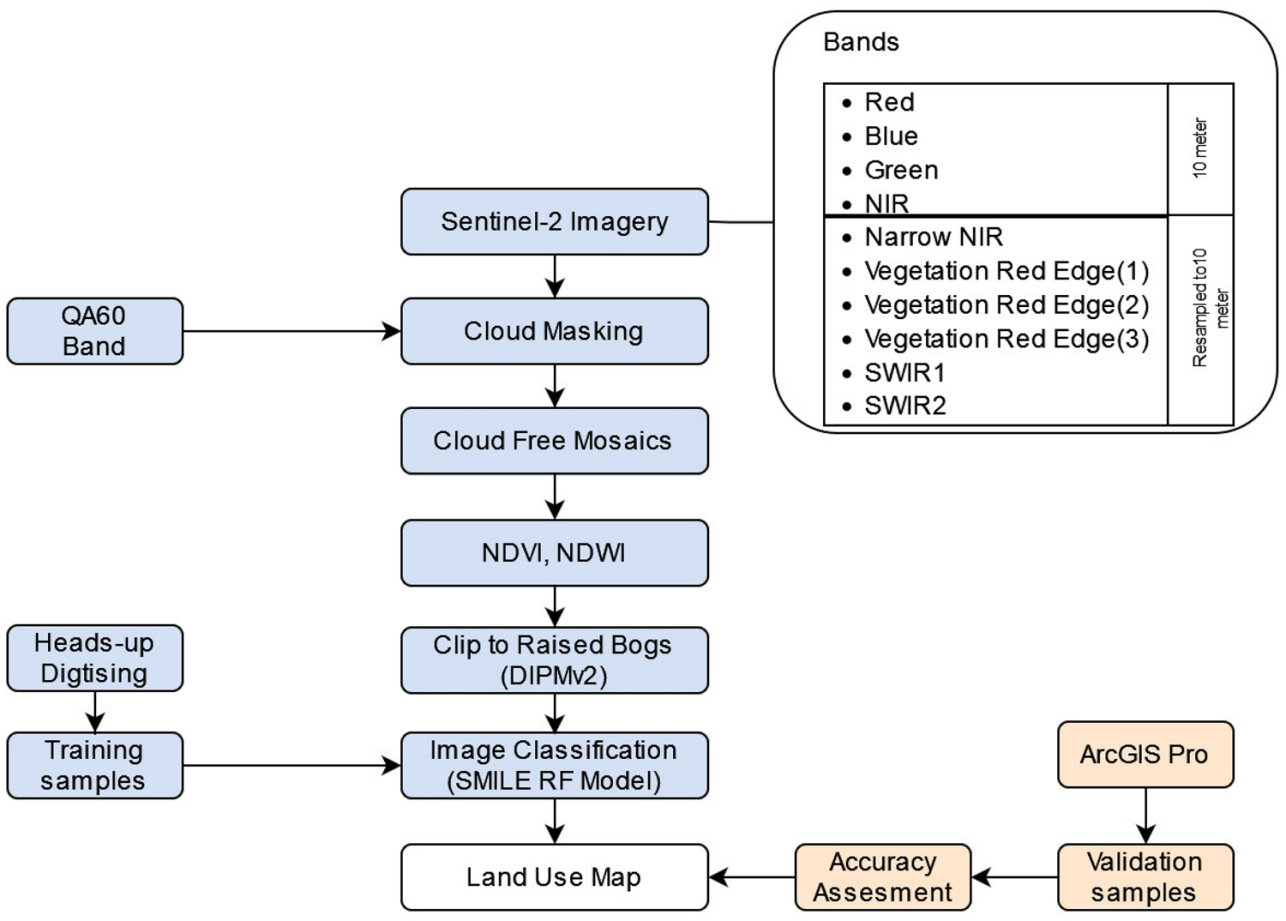
Data processing workflow. NIR (Near Infrared), SWIR (Shortwave Infrared), NDVI (Normalised Difference Vegetation Index), Normalised Difference Water Index (NDWI), QA60 (Quality Assessment) band for cloud and cirrus masking, and DIPMv2 (Derived Irish Peat Map version 2).

A remote sensing image classification-based approach was used in this study and implemented in GEE and ArcGIS Pro desktop version 2.7 (ArcPro from here onwards). The approach consisted of accessing, pre-processing, and classifying land use using LUCIP in GEE. Accuracy assessment was conducted using ArcPro. The imagery was accessed on a national scale and constrained to Irish-raised bogs using the DIPMv2. An overview of this process is shown in Fig. 1.

### Image pre-processing

Despite the high temporal resolution (five days) of Sentinel-2 (A and B), obtaining a cloud-free mosaic for wall-to-wall coverage of raised bogs in Ireland was challenging. To address this, it is important to develop an efficient cloud removal methodology. Accordingly, a temporal filter was applied to obtain imagery over three years (1st January 2018 – 31st December 2020). A selection criterion to obtain images with less than 10% cloud cover was also applied; a percentage greater than this would return cloud-contaminated images that were not useful for analysis. Based on these criteria, 1483 images were available. The remaining cloudy pixels were masked out using a cloud masking function (*masksS2clouds)* which is based on the ‘QA60’ quality flag band used for cloud and cirrus. Finally, the images were stacked together to generate a single cloud-free composite using the median (*ee.reducer*) composite function available in GEE. This also helped eliminate pixels with extreme values, thus removing the remaining artefacts. The final image was composed of pixels with minimal or no cloud cover.

### Training sample data

The training and validation data used in this study were independent of each other. The training data was based on randomly generated sample polygons created within GEE for each land use class (Table 2). Validation sample points were generated using ArcPro. A classification schema (LUCIP) was developed to identify seven land-use classes: cutover, cutaway, grassland, forestry, remnant peatlands (high bog), and built-up/infrastructure areas (Table 2).

**Table 2:** Description of land use classes based on Land Use Classification for Irish Peatland (LUCIP) developed in this study.

Land use class	Description
Cutaway	Land that has been subjected to industrial peat extraction, with the peat removed and/or left with a thin layer of soil, managed by BnM and other private companies with large-scale mechanised peat extraction.
Cutover	Land that has been subjected to domestic peat extraction, hand-cut, small-scale mechanised, includes bare peat, interspersed woodland, heath, and scrub.
Grassland	Agriculture grassland that is used for pasture or hay, silage, and grazing.
Forestry	Land covered with trees; afforested areas are mostly covered by evergreen tree species
Remnant Peatland	Land that has a high percentage of peat (near natural and high bog areas,) and is characterised by the presence of sphagnum mosses and other bog flora, not directly affected by human intervention. Revegetated areas of post-extraction activities.
Water bodies	Land covered with water, including natural waterways and surface water on raised bogs that have appeared after rewetting activities and/or abandonment.
Built-up/Infrastructure	Land used for human settlements and infrastructure, such as buildings, roads, windfarms etc.,

The collection of training data was based on the visual interpretation technique, which was used by an expert operator to generate randomly distributed sample polygons for each land use class^50^. The training data consisted of 366 polygons (148405 pixels) that were randomly distributed across the study area (Table 3).

**Table 3:** Characteristics of randomly distributed training and validation sample data.

Land use class	No. of training polygons	No. of training pixels (sum per training polygon)	No. of validation points
Cutaway	37	94,672	197
Cutover	45	4345	130
Grassland	32	7320	660
Forestry	100	11,627	252
Remnant Peatland	62	28,038	121
Water bodies	64	1816	50
Built-up/Infrastructure	26	587	50
Total	366	148,405	1460

**Figure 2:**
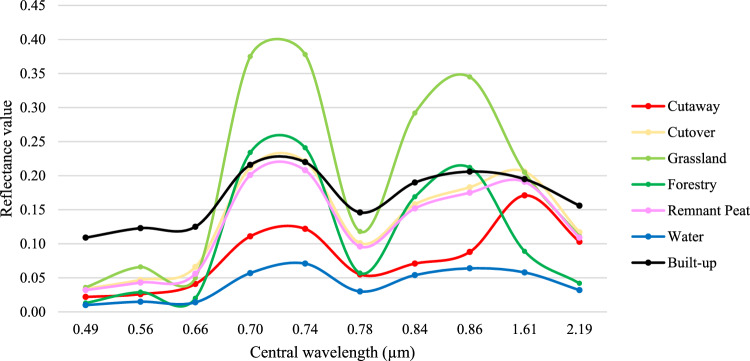
Spectral reflectance of the land use types across the ten spectral bands (Sentinel-2).

The spectral signature of each land use class is shown in Fig. 2. The mean reflectance values across multiple Sentinel-2 bands demonstrate significant spectral separability in the visible and NIR range i.e., 0.49 to 0.78 µm. Notably, built-up areas exhibit high reflectance, whereas water has low reflectance, making them significantly distinguishable. Cutaway and grassland show high reflectance in the NIR region. Cutover and remnant peatlands, however, may exhibit some similarities, given that the cutover class includes dynamic vegetation environment. The spectral separability for most classes diminishes for longer wavelength bands, specifically at 1.61 and 2.19 µm. Nevertheless, differences in spectral reflectance between all the classes remain prominently discernible, underscoring the effectiveness of the sampling procedure employed for land use classification.

### Classification of satellite imagery

The random forest classification model, an ensemble classifier^51,52^, was trained using training data. The random forest algorithm is based on an ensemble of several decision trees, each with multiple nodes^51^. Classification was implemented using the GEE platform. A slightly improved version of the random forest model known as the Statistical Machine Intelligence and Learning Engine (SMILE) random forest algorithm was used in this study^53^. The classification model was trained using the training data, with the number of variables and number of trees being the two critical parameters. The optimal values of these parameters were determined using the square root of the number of features and trial-and-error method. Based on these procedures, the optimal number of trees was determined as 20. Each tree in the model consists of multiple nodes, and each pixel is assigned a label by each tree. The final label for each pixel is determined by aggregating the labels assigned by all trees through a majority vote^51^. The random forest model and its optimisation procedures were implemented using the GEE. The variable importance for all spectral bands, NDVI, and NDWI were also assessed. Spectral reflectance bands (2, 5, 12, and 11) were ranked higher, while NDVI and NDWI were ranked lower (see Supplementary Fig. S1 online). The accuracy of the final output map was assessed using the validation data.

### Validation (accuracy assessment)

After conducting a qualitative accuracy assessment (visual evaluation), the final map was downloaded from GEE and imported into ArcPro. An overall quantitative accuracy assessment was conducted using ArcPro, with independent validation point sample data (Table 3). Yelena and Antonia^54^ emphasise the importance of using higher-quality reference data derived through a sampling approach to assess the accuracy of land use maps. They further noted that the data could be based on field sampling or a higher resolution spatial resolution aerial imagery. The latter approach was used in this study since the first approach could be laborious and time-consuming for national scale mapping. The “create accuracy assessment tool” in ArcPro was used with a stratified random sampling strategy^55^. The tool generates random points, and the sample size is based on the proportion of the area for each class. This process ensures that a sufficient number of reference samples is created for each stratum (class)^55^. A total of 1460 points were obtained (Table 3). Each validation sample data point was assessed and manually labelled through the visual interpretation of very high-resolution aerial imagery (25 cm) by a single expert operator. After completion of manual labelling, a confusion matrix for accuracy assessment was generated using the “compute confusion matrix” tool in ArcPro. This was performed by comparing the classes obtained using the classification model with the ground truth data. Multiple statistics were derived from the confusion matrix to quantify the map accuracy. These include the Overall Accuracy (OA), overall agreement between classification (map), and ground truth for all classes. The User's accuracy (UA) measures the agreement between the classified pixels and the ground-truth data for a specific class from the user's perspective, whereas the Producer's accuracy (PA) measures the agreement between the classified pixels and the ground-truth data for a specific class from the producer's perspective^56^. Lastly, owing to the presence of errors in the classification results, a simple pixel counting method to calculate the land use area is not adequate. Therefore, ‘good practices’, as suggested by Olofsson et al.^55^ were used to generate an area-based error matrix and calculate the unbiased land-use area for each class.

### CO_2_ emission calculations

**Figure 3:**
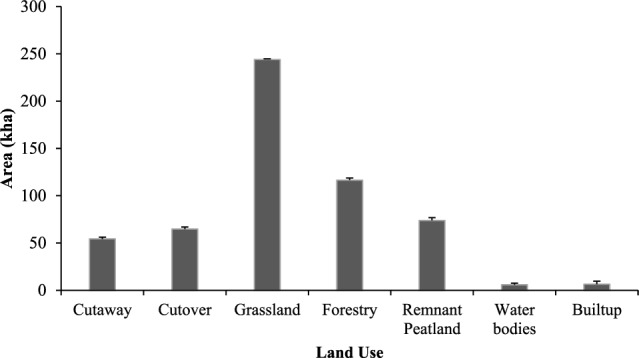
Land use area in kilo hectares (kha) for each class, error bars for standard errors in kha.

CO_2_ emissions were calculated using the IPCC T1 EFs and literature-based T2 EFs using Eq. (3). Four dominant land use classes (cutaway, cutover, forestry, and grassland), which cover ~ 85% of the total raised bog land use area (Fig. 3) were used for emission estimation. For the remaining classes (remnant peatlands, water bodies, and built-up areas), neither T1 nor T2 emission factors were available; therefore, CO_2_ emissions could not be estimated. T1 default EFs from the IPCC Wetland Supplement (2013) and T2 EFs by Aitova et al.^26^ which are based on case studies in Ireland for grasslands, cutover and cutaway sites were used. The EF for forestry is based on eight drained and afforested peatland sites in Ireland^57^.

3 $${\text{CO}}_{{2}} \;{\text{emission }}\left( {{\text{tCO}}_{2} - {\text{C}}\;{\text{ha}}^{ - 1} {\text{y}}^{ - 1} } \right) = {\text{land}}\;{\text{use}}\;{\text{area}}\;\left( {{\text{ha}}} \right) \, \times {\text{ EF }}\left( {{\text{tCO}}_{2} - {\text{C ha}}^{ - 1} {\text{y}}^{ - 1} } \right)$$

where *t* is tons, ha is hectares, *y* represents a year, and EF is the land use specific emission factor.

## Results

The statistics for the area of each land use class in raised bogs obtained using the error matrix (see Supplementary Table S1 online) are presented in Fig. 3. Almost half of the raised bogs mapped in this study are covered by grassland i.e., 43% (244,100 ha). Forest covers about 21% (116,427 ha), cutaway 10% (54,302 ha) and cutover cover 11% (646,99 ha). Remnant peatlands (high bog) area covers about 13% (73,795) ha. Water bodies and built-up areas account for about 1% (1542 ha and 1358 ha, respectively). The water bodies here constitute both natural waterways included in the coarse resolution DIPMv2 and surface water on raised bog appearing possibly after rewetting activities.

**Figure 4:**
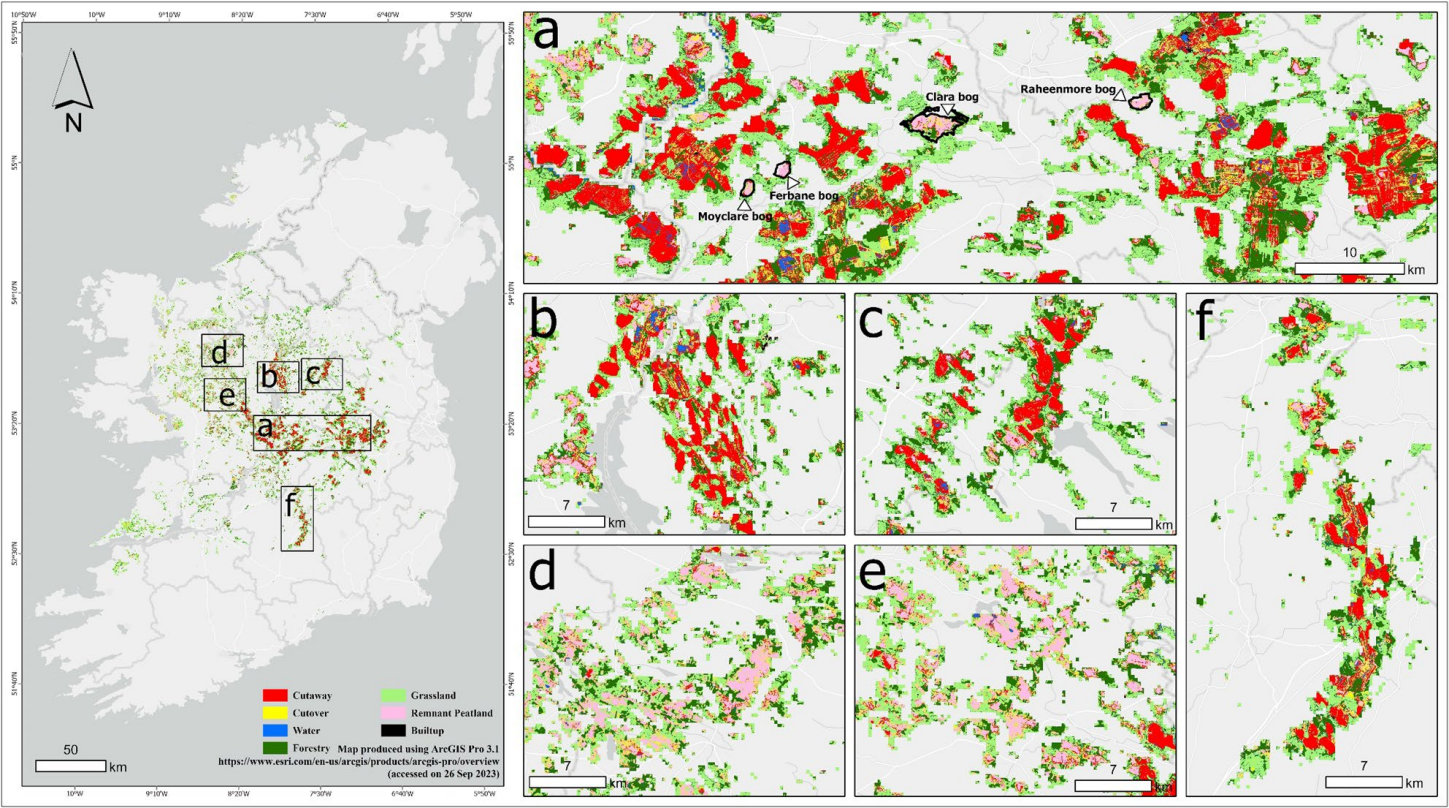
Land use on raised bogs. (**a**), (**b**), (**c**) and (**f**) are dominated by industrial peat extraction sites (mostly Bord na Móna), whereas (**d**) and (**e**) have a mix of remnant peatland, cutover, grassland, and forest.

The prevalence of land use activities can be seen across all raised bogs in the midlands of Ireland Fig. 4. Industrial peatland extraction sites are primarily located in the midlands (Fig. 4a) and some parts of the north (Fig. 4b and c) and south (Fig. 4f) of the midlands. Approximately 65% of these industrial extraction sites are owned by BnM. The other 35% constitute non-BnM peat extraction carried out on an industrial scale similar to BnM (Fig. 4d and e). BnM landholding and Special Area of Conservation (SAC) boundary data were used to further examine the distribution of land use to better understand the status and condition of raised bogs under different management regimes, i.e., industrial and protected (Table 4). Additionally, Coillte landholding boundary data was also used to assess land use. It owns ~ 31,000 ha of raised bog areas accounting for about one-third of the total forestry on these bogs.

**Table 4:** Proportion of each land use on Bord Na Mónaa (BnM) and Special Area of Conservation (SAC) under the national parks and wildlife services (NPWS). All areas are in hectares (ha).

Class	BnM (ha)	SAC (ha)
Cutaway	42,264	2460
Cutover	7303	4907
Grassland	2379	7318
Forestry	9067	3490
Remnant Peatland	6491	13,053
Water bodies	1580	1340
Built-up/Infrastructure	388	197
Total	69,472	32,568

The exposed peat on BnM landholdings amounts to around 42,264 ha. The rest of the cutaway bogs, totalling about 2,460 ha, are in SAC, highlighting the existence of a substantial amount of bare peat on these “protected” bogs with active cutting also taking place^58^. Cutaway bogs in midlands have interspersed forestry (Fig. 4a). The areas on the edges of these bogs represent agricultural grasslands. The remnant peatlands in the midlands and are mostly on SAC sites such as Clara Bog, Ferbane Bog, Moyclare Bog, and Raheenmore Bog (Fig. 4a). Overall, there is about 13,053 ha of the remnant peatlands located on the SAC, which is approximately 1% of the total peatland area and most of the “actively forming raised bogs” are possibly located in these sites^59^. The majority of the remnant peatlands are in the west of the midlands and are surrounded by peat-extraction activities (Fig. 4d and e). Lastly, the scattered water bodies on cutaway sites in the Midlands and some parts in the North are possible signs of some of the rewetting activities being carried out by BnM (Fig. 4a and b).

### CO_2_ emissions

CO_2_ emissions presented in Table 5 are calculated using Eq. (1) for the four land use classes. The area (ha) is estimated from the land use map (Fig. 4). T1 emissions were ranging between 303,710 and 1,293,730 t CO_2_-C ha^−1^ y^−1^ with grassland being the highest emitters followed by forestry, cutover, and cutaway. T2 emissions were in the range of 65,705–317,330 t CO_2_-C ha^−1^ y^−1^, with the highest emissions in grassland and the lowest emissions in cutaway. The T1 emissions for grassland were four-fold higher than T2 emissions. The total CO_2_ emissions from Irish-raised bogs based on the four land use classes mapped here account for 0.68 Mt CO_2_-C ha^−1^ y^−1^ as per T2 and 1.92 Mt CO_2_-C y^−1^ as per T1.

**Table 5:** Emissions from four dominant land use classes, with Intergovernmental Panel on Climate Change (IPCC) Tier 1 and 2 (T1 and T2) Emission Factors (EFs) with 95% confidence intervals values are shown in brackets, Emissions based on the area in hectares (ha) and EFs (in tonnes of CO_2_-C per hectare per year).

Land Use	Area (ha)	T1 (IPCC) EFs	T1 Emissions (t CO_2_-C ha^−1^ y^−1^)	T2 (literature) EFs	T2 Emissions (t CO_2_-C ha^−1^ y^−1^)	No. of sites for T2 EFs	References
Cutover	64,699	2.8 (1.1–4.2)	181,157	1.21 (0.4 – 2.0)	102,871	3	^26^
Cutaway	54,302	2.8 (1.1–4.2)	152,046	1.59 (1.2 – 2.0)	65,705	4	^26^
Forestry	116,427	2.6 (2.0–3.3)	302,710	1.68 (1.04 – 2.32)	195,597	8	^57^
Grassland	244,100	5.3 (3.7–6.9)	1,293,730	1.3 (0.04 – 2.55)	317,330	3	^26^

### Accuracy assessment

The OA of the final land use map was 89% which is computed from the diagonal line of the confusion matrix (Table 6), representing the number of correctly classified classes. A total of 1303 of 1460 points were correctly classified. The UA ranges from 70% (water and cutover) to 96% (forest), and PA ranges from 64% (built-up) to 97% (cutaway).

**Table 6:** Result of accuracy assessment for the seven land use classes, including Overall Accuracy (OA), User's Accuracy (UA), and Producer's Accuracy (PA).

Class	A	B	C	D	E	F	G	Total	UA (%)
Cutaway (A)	**164**	12	1	8	2	10	0	197	83
Cutover (B)	0	**91**	0	4	15	19	1	130	70
Water (C)	1	0	**35**	8	2	0	4	50	70
Forest (D)	1	2	0	**244**	4	1	0	252	96
Grassland (E)	2	1	1	12	**624**	6	14	660	94
Remnant Peatland/High Bog (F)	1	2	0	2	5	**111**	0	121	91
Built-up (G)	0	0	0	3	13	0	**34**	50	68
Total	169	108	37	281	665	147	53	**1303**	**OA (%)**
PA (%)	97	84	94	86	93	75	64		89.2

The emboldened diagonal elements represent accurately classified areas.

The results show the highest PA for cutaway (97%) and grassland classes (93%) while remnant peatlands (75%) and built-up (64%) have the lowest PA. Forestry (97%), grassland (94%) and remnant peatlands (91%) all show high UA, while built-up (68%), cutover and water (70%) show low UA. The lower UA of the cutover class (70%) can be attributed to the presence of heterogeneous vegetation, resulting in a mosaic landscape following the abandonment of extraction sites. As a result, this class is prone to misclassification, particularly with remnant peatlands (another landscape characterised by heterogeneity) (Fig. 2). The lower UA of water bodies (70%) and misclassification with forestry can be attributed to overlap in spectral signatures in partially vegetated and surface water areas, as shown in Fig. 4.

## Discussion

The degradation of Irish-raised bogs due to centuries of human exploitation is widely acknowledged^6,12,13^. Nevertheless, a significant knowledge gap exists in the absence of “robust aerial data” for accurate assessment of land use on the raised bog^14,26^. This gap in spatial data is addressed through the development of the LUCIP taxonomy and its implementation in GEE using Sentinel-2 data. This study provides the first high-resolution wall-to-wall coverage of land use on raised bogs in Ireland. The robust methodology presented here facilitates an accurate assessment of the magnitude and extent of land use and CO_2_ emissions on these ecosystems. It utilises cloud computing (GEE), temporal mosaicking, and machine learning (random forest and high-resolution remote sensing imagery (Sentinel-2)) to overcome the issue of persistent cloud cover in Ireland. The accuracy assessment results (overall accuracy of 89%) showed good agreement between the map and the reference data.

Currently, the Irish National Inventory Report (NIR) only considers peat extraction activities (industrial/domestic) in the “managed wetlands” category for all peatlands (blanket and raised bogs). This area is reported as 70,020 ha, of which 400 ha is domestic cutover with the remainder being industrial cutaway, and emissions calculations in the NIR are also reliant on these area estimates^32^. In literature, this figure is ~ 80,000 ha, which is mainly the BnM landholding^12,14,32,60^. However, the findings of our study which focused only on raised bogs, indicate that peat extraction (cutaways and cutovers) is considerably more prevalent than currently reported and extends to approximately 119,000 ha. This is 70% higher than the NIR-reported “managed wetland” area figures for all peatlands. Hence, we address the overall ambiguity in exisiting land use estimates by providing accurate spatially explicit land use information derived from robust remote sensing methods.

The results depict heterogeneous land use in raised bogs across Ireland. The midland raised bogs (Fig. 4a, b, c, and f) are dominated by industrial mining activities as well as conversion to grassland and forestry. The more peripheral raised bog areas to the west, northwest, and southwest of the midlands are dominated by conversion to grassland and forestry. In general, the most extensive land use on raised bogs is agricultural grassland which is distributed across the region. Grasslands mainly occur on the margins of cutaway, cutover and remnant peatlands. The remnant peatland class, which represents the remaining areas of the raised bogs, are not in pristine condition, and are more abundant towards the North and West of the midlands, away from the areas of intensive industrial peat extraction activities. The BnM Peatland Climate Action Scheme (PCAS) aims to restore degraded peatlands within BnM landholding and is a good start for mitigation and adaptation, which are more intact raised bogs in the north and west that could be targeted for active restoration by policymakers, facilitating carbon retention in these ecosystems. It is also pertinent to mention that the PCAS was initiated at some of the former industrial peat extraction sites during the timeframe of this study. Most of these areas are still bare peat which means the data produced in this study could be used as baseline data for tracking and monitoring the PCAS over time. Overall, the maps show that the current level of human-induced degradation of these raised bogs through land-use change requires immediate action for sustainable management of these ecosystems.

**Figure 5:**
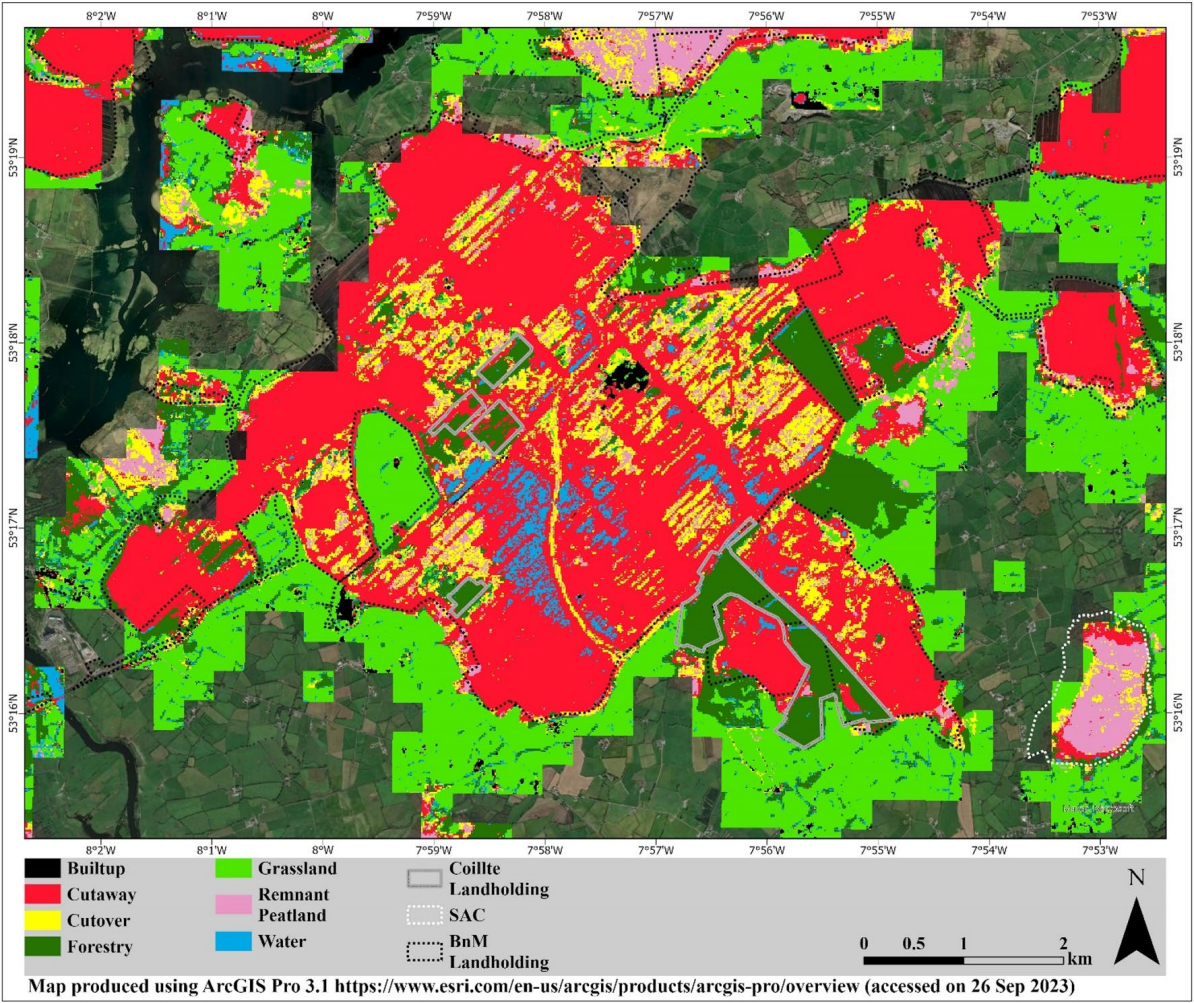
Typical example of a former industrial bog complex mapped in this study. The areas outlined by the black dotted lines are within the BnM (Bord na Móna) landholdings, whereas the white dotted lines in the southeast section of the map show a bog under SAC (Moyclare Bog). The grey boundaries in the BnM landholdings and outside of it are Coillte landholdings.

On BnM landholdings, industrial extraction activities have gradually ceased over the past two decades, with an announcement of complete cessation in 2021. The results of this are beginning to be observed in the data. At the Blackwater site (Co. Offaly) site (Fig. 5), areas are classified as remnant peatlands, which is an indication of revegetation after the cessation of extraction activities (pre-2000) and subsequent rewetting in 1999^61^. These sites are going through a transformation with land use conversion to forestry or a diverse mosaic of vegetation communities composed of heather, shrubs, grasses, and interspersed larger plants. The forestry areas in Fig. 5, are on Coillte landholdings and are highlighted with a grey outline. Other areas with surface water are possibly signs of rewetting activities and are represented by surface water here (Fig. 5). Vegetation cover at Blackwater has increased over the years, albeit slowly, with Sphagnum mosses and other bog species recolonising the area i.e., sparse remnant peatlands patches among the prevalent bare peat (cutaway) (Fig. 5). This recovery process is not only important for carbon sequestration and climate change mitigation but also for the restoration of the unique biodiversity and ecosystem services provided by peatlands. An SAC site is outlined (white dotted line) in the bottom right (Fig. 5) and shows a good example of what is considered a “near natural” raised bog e.g., intact centre but degraded margins where substantial cutting has taken place and areas converted to grassland. These changes can be seen using Sentinel-2 which demonstrates the utility of this methodology for tracking and monitoring land use change in raised bogs over time.

The EU has established a range of regulations and initiatives that directly or indirectly require restoration and sustainable management of peatlands. These include the Habitats Directive (Council Directive 92/43/EEC), Water Framework Directive (Directive 2000/60/EC), EU Biodiversity Strategy for 2030, Common Agricultural Policy (CAP), Natura 2000 Network (Directive 2009/147/EC), LIFE Programme, EU Climate Adaptation Strategy, and more recently the Nature Restoration Law which includes restoration of peatlands by binding targets and net zero CO_2_ emissions from these ecosystems by 2050. The effective implementation of these initiatives is only possible through robust mapping, tracking, and monitoring^62^ methodologies such as those proposed in this study.

The map produced here serve as an important indicator for determining the baseline status and condition of these ecosystems as well as providing a quantification of CO_2_ emissions hotspots. By providing a detailed map of BnM/non-BnM industrial activities and domestic extraction activities, this study not only highlights these activities in a spatial context but also estimates emissions from them. The emissions calculated in this study based on IPCC T1 and Ireland-specific T2 EF show a substantial difference. The IPCC default emission factor is higher than the Irish T2 EF resulting in higher emission estimation. One of the limitations of using IPCC T1 EFs is that these EF are based on limited case studies with diverse geographical locations, climatic conditions and ecology not necessarily suited to Irish Peatlands. The EFs proposed by Aitova et al.^26^ and the EFs from the study by Jovani‐Sancho^57^ are based on measurements from specific sites in Ireland, the United Kingdom and Germany, and may not be a true representatives of other sites in the country. For example, T1 EFs for grasslands (5.3 t CO_2_-C ha^−1^ y^−1^) are mostly based on study sites from Germany, which are under more intensive management practices compared to Ireland^26,63^. This may lead to a substantial difference when comparing T2 EFs for grasslands in Ireland (1.30 t CO_2_-C ha^−1^ y^−1^)^26^. While these EFs could be refined specifically for Ireland, their use by Aitova et al.^26^ is a significant improvement compared to the T1 EFs. Finally, the delineation of raised bogs in this study relies on an existing peatland extent map i.e., DIPMv2. Although, DIPMv2 is most current peatland map in Ireland, with an overall accuracy of 88%, it tends to underestimate the presence of peatlands with areas smaller than 7 ha^5^. This study can be expanded to these missing areas if the DIPMv2 is updated in the future.

Nevertheless, this study demonstrates the robustness and utility of remote sensing methods to accurately map the land use on peatlands and to integrate these data with the latest T2 EFs thus refining estimates of CO_2_ emissions from different land use on Irish peatlands. The LUCIP implemented in GEE facilitates the development of highly accurate land use maps that can aid the refinement of national-scale T2 reporting in these globally rare ecosystems. These integrated spatial datasets can help inform decision-making for sustainable land management practices and conservation. Furthermore, detailed habitat mapping of the remnant peatland class using a high-resolution dataset and integrated Sentinel-1 and 2 approach could be useful for monitoring active raised bog areas in SAC^42,64^. Future work using the LUCIP could address the large knowledge gap regarding land use type and extent on blanket bogs, which account for ~ 70% of peatlands in Ireland and have not been studied at this level in Ireland. It is important that the EFs and emissions are assessed for these areas to identify C and GHG emission hotspots and areas for targeted restoration.

## Conclusion

In this study, a spatially explicit dataset of land use on Irish raised bogs was created by integrating the DIPMv2 and Sentinel-2 satellite images collected between 2018 and 2020. Overall, the accurate results (OA = 89%) of this study provide valuable insights into the spatial extent of land use in raised bogs in Ireland. These data were integrated with T2 EFs to refine the estimation of CO_2_ emissions from the four major land classes on raised bogs. These spatial data on land use can inform policies on land use and emissions at a national scale. The map also enhances the current understanding of the extent and scale of different land uses (especially peat-extraction activities) in a spatial context. The state and condition of raised bogs in Ireland present a pressing concern due to their substantial CO_2_ emissions. Urgent measures are required to address this issue, including (i). mitigating emissions and (ii). implementing sustainable management practices to promote carbon sequestration within these ecosystems and prevent additional degradation. This spatial information can be used to inform the development of more sustainable approaches to peatland management in the country, the decision-making process for developing such policies and effective strategies to mitigate these management impacts.

### Supplementary Information

Supplementary Information 1.

Supplementary Information 2.

## Data Availability

The datasets used and/or analysed during the current study are available from the corresponding author upon reasonable request.
