# Supplementary material for: Quantifying peatland land use and CO2 emissions in Irish raised bogs: mapping insights using Sentinel-2 data and Google Earth Engine
Source: Sci Rep. 2024 Jan 12;14:1171. doi: 10.1038/s41598-024-51660-0 (PMC10786884; doi:10.1038/s41598-024-51660-0)
Supplement: Supplementary file 1 — Supplementary Information 1. [file 41598_2024_51660_MOESM1_ESM.docx]

**Quantifying Peatland Land Use and CO_2_ Emissions in Irish Raised Bogs: Mapping Insights using Sentinel-2 data and Google Earth Engine**

**(Supplementary Material)**

Wahaj Habib ^a *^, Ruchita Ingle ^b, c^, Matthew Saunders ^b^ and John Connolly ^a^

a) Discipline of Geography, School of Natural Sciences, Trinity College Dublin, Ireland

b) Discipline of Botany, School of Natural Sciences, Trinity College Dublin, Ireland

c) Water Systems and Global Change Group, Wageningen University, Wageningen, The Netherlands

**Supplementary Figure for section 2.5**

Supplementary Figure S1: Variable importance in the Random Forest model trained with variables from 10 spectral reflectance bands, NDVI and NDWI

**Supplementary Table for section 3**

Supplementary Table S1: Unbiased area estimates, the table also presents standard error and 95% Confidence Interval values for each class

| **Area-based Error Matrix** | | | | | | | |
| --- | --- | --- | --- | --- | --- | --- | --- |
| **Classified** | **Cutaway** | **Cutover** | **Water** | **Forest** | **Grassalnd** | **Rem. Peat** | **Builtup** |
| Cutaway | 0.0989 | 0.0072 | 0.0006 | 0.0048 | 0.0012 | 0.0060 | 0.0000 |
| Cutover | 0.0000 | 0.0553 | 0.0000 | 0.0024 | 0.0091 | 0.0115 | 0.0006 |
| Water | 0.0003 | 0.0000 | 0.0096 | 0.0022 | 0.0005 | 0.0000 | 0.0011 |
| Forest | 0.0008 | 0.0016 | 0.0000 | 0.1973 | 0.0032 | 0.0008 | 0.0000 |
| Grassland | 0.0014 | 0.0007 | 0.0007 | 0.0084 | 0.4351 | 0.0042 | 0.0098 |
| Remnant Peatland | 0.0009 | 0.0019 | 0.0000 | 0.0019 | 0.00 | 0.1050 | 0.0000 |
| Builtup | 0.0000 | 0.0000 | 0.0000 | 0.0006 | 0.0026 | 0.0000 | 0.0068 |
| **Total Estimated Area Proportions** | 0.102 | 0.067 | 0.011 | 0.218 | 0.456 | 0.128 | 0.018 |
| **Class Area Estimates (ha)** | 54301.80 | 35385.73 | 5786.48 | 115458.44 | 242159.53 | 67674.29 | 9705.63 |
| **Standard Error of Area Estimates** | 0.00356 | 0.004222758 | 0.00129 | 0.00418 | 0.00545 | 0.00308 | 0.00617 |
| **Standard Error of Area Estimates (ha)** | 1886.37 | 2240.05 | 682.93 | 2217.80 | 2889.98 | 1635.13 | 3274.76 |
| **95% Confidence Interval (ha)** | 3697.28 | 4390.51 | 1338.55 | 4346.89 | 5664.36 | 3204.86 | 6418.53 |
